# Splenic vein rupture in pregnancy: A case report and systematic review of the literature

**DOI:** 10.1002/ijgo.15842

**Published:** 2024-08-01

**Authors:** Daniel Kane, John M. Keaney, Elizabeth Tunney, Ronan Daly, Emma Doyle, John B. Conneely, Conan McCaul, Michael Geary, Jennifer Donnelly, Claire M. McCarthy

**Affiliations:** ^1^ Department of Obstetrics & Gynaecology Royal College of Surgeons in Ireland, Rotunda Hospital Dublin 1 Ireland; ^2^ School of Medicine, Royal College of Surgeons in Ireland, St Stephens Green Dublin 2 Ireland; ^3^ Department of Pathology Rotunda Hospital Dublin Ireland; ^4^ Department of Surgery Mater Misericordiae Hospital Dublin Ireland; ^5^ Department of Anaesthesia Rotunda Hospital Dublin Ireland; ^6^ Department of Obstetrics and Gynaecology Rotunda Hospital Dublin Ireland; ^7^ School of Medicine, University College Dublin Dublin Ireland

**Keywords:** aneurysm, obstetric, pregnancy, splenic vein aneurysm, systematic review

## Abstract

Splenic vein aneurysm (SVA) rupture is a rare clinical entity, with few case reports detailing its occurrence during pregnancy. We describe a case of a SVA rupture and present a systematic review of the literature in relation to splenic vein rupture, with or without aneurysm. Our case was of a 30‐year‐old woman, Para 4 at 37 weeks' gestation who presented with significant abdominal pain and subsequent maternal collapse. Massive intra‐abdominal hemorrhage was identified, with splenic vessel rupture suspected. A splenectomy and partial pancreatectomy were performed along with massive blood product transfusion. There was both maternal and fetal survival with no long‐term sequelae at follow‐up. Histological examination of the spleen and its vessels noted a SVA rupture. In a subsequent systematic review of the literature, we identified 10 cases of splenic vein rupture with only two previously documented cases of SVA rupture in pregnancy. Maternal and fetal survival has only been reported in two cases of splenic vein rupture, with ours being a third.

## Synopsis

Splenic vein aneurysm (SVA) rupture is a rare clinical entity, with few case reports detailing its occurrence during pregnancy.

## 1 INTRODUCTION

Spontaneous intra‐abdominal hemorrhage (SIAH) in pregnancy presents a significant threat with high rates of fetal mortality and maternal morbidity.^1^ This condition refers to non‐traumatic bleeding within the abdominal cavity that occurs spontaneously during pregnancy, labor, or in the postpartum period, without direct obstetric interventions or trauma. The etiology of SIAH in pregnancy is broad, encompassing various conditions, ranging from obstetric causes such as uterine rupture to non‐obstetric causes such as rupture of parenchymal organs (such as the liver, spleen, or kidney) and rupture of abdominal or pelvic vasculature, which includes aneurysmal rupture.^2^ Among these non‐obstetric causes, the rupture of splenic vessels, specifically the splenic artery, though uncommon, is well‐documented in the literature.^3^ However, the rupture of the splenic vein either spontaneously or due to aneurysm has rarely been reported. Pregnancy, marked by profound hemodynamic and hormonal changes, poses unique challenges to the maternal vascular system, increasing the predisposition to a spectrum of vascular complications. Clinical manifestations of intra‐abdominal bleeding in pregnancy are non‐specific, including pain, nausea, maternal collapse, or evidence of fetal compromise, which may overlap with other more common pregnancy‐related complications such as placental abruption or uterine rupture. Therefore, the prompt recognition and management of this condition presents significant clinical challenges, necessitating a high index of suspicion and a multidisciplinary approach once identified.

In this paper, we present a case of splenic vein aneurysm (SVA) rupture in the third trimester of pregnancy preceded by maternal collapse. Additionally, we conducted a systematic review of splenic vein rupture during pregnancy and the postpartum period to allow for comparison and to contrast our case with other reported cases. This effort aims to enhance understanding and awareness of this rare but critical complication in pregnancy, facilitating early recognition and management to improve maternal and fetal outcomes.

## 2 METHODS

### 2.1 Systematic review and case review

The electronic database search included Medline (1946 to March 2024) and Embase (1959 to March 2024). Key MESH and Boolean terms were used for the search. Articles were limited to those available in English. PRISMA was used to assess publications for inclusion in the review. Initial searches showed 146 articles, from which nine full‐text papers were obtained for detailed analysis. One further paper was obtained by citation search. The 10 papers included in this review report a total of 10 pregnancies involving splenic vein rupture; two report SVA, bringing three cases published in the literature on the inclusion of our case. Hospital notes were retrospectively reviewed for the case we discuss.

### 2.2 Ethical considerations

This article does not contain any studies with human participants or animals performed by any of the authors. Written informed consent from the patient for a case report was obtained. Ethical approval is not required from our institution for case reports.

### 2.3 Case review

We describe the case of a 30‐year‐old woman, in her sixth pregnancy at 37 weeks and 5 days gestation who was brought by ambulance to our obstetric emergency department with a 1‐day history of worsening abdominal pain. Her obstetric history was of four term spontaneous vaginal births following uncomplicated pregnancies. She had previously had a first‐trimester missed miscarriage which had been managed medically with no complications.

As regards the index pregnancy, she had booked at 12 weeks' gestation, with a trans‐abdominal ultrasound at this time demonstrating a singleton viable intrauterine pregnancy. She was serology‐negative and rubella‐immune. Her past medical history included urolithiasis, with recurrent urinary tract infections during this index pregnancy with a recent admission for pyelonephritis and stent insertion on the right side. She had a body mass index of 37 (calculated as weight in kilograms divided by the square of height in meters). In relation to this presentation, the patient reported that the abdominal pain was left‐sided, constant, with associated vomiting. She denied symptoms of vaginal bleeding or spontaneous rupture of membranes. There was no history of any trauma.

On initial assessment she was hemodynamically stable with a heart rate of 99 beats/minute (bpm), a blood pressure of 138/64 mm Hg, oxygen saturations of 100% on room air, temperature of 36.5°C and a respiratory rate (RR) of 19 breaths/minute. On abdominal examination, it was noted that there was tenderness on palpation of the left side of her abdomen, with no rebound or rigidity. There was no renal angle tenderness noted. A bedside trans‐abdominal ultrasound was performed that showed a single intrauterine pregnancy, with both a fetal heartbeat and fetal movement visible, with a normal liquor volume noted. There was no comment regarding intra‐abdominal free fluid. Fetal presentation was breech. On vaginal examination her cervix was posterior, long and closed. She was admitted to the antenatal ward for observation and analgesia.

Six hours into her admission she reported to the midwifery staff that her abdominal pain was worsening and she was feeling faint. An urgent review was carried out by the obstetric and anesthetic team. Her heart rate remained normal (87 bpm), but she was significantly hypotensive (58/38 mm Hg). A point of care abdominal ultrasound noted a fetal bradycardia (<60 bpm). A decision was made to perform an emergency cesarean section with a presumptive diagnosis of a placental abruption recorded. The patient underwent a rapid sequence induction general anesthesia.

On opening the abdomen, significant hemoperitoneum was noted with an intact uterus. The on‐call surgical team from the affiliated tertiary unit was asked to attend immediately. The hysterotomy was performed and a live‐born female infant was delivered with Apgar scores of 1, 4, 6, and 7 at 1, 2, 5 and 10 min of life, respectively. The infant was successfully resuscitated and underwent therapeutic hypothermia. The initial low transverse abdominal incision was extended to a midline laparotomy. The on‐call general surgeon reviewed the patient intra‐operatively and it was noted that there was a hematoma in the lesser sac in the left upper quadrant, which appeared to be mesenteric in origin. Systematic examination of all organs was undertaken and no bleeding point was identified; hemostasis was confirmed. The patient was then transferred to the recovery room.

Two hours postoperatively in the recovery room, the patient became acutely unwell for a second time, with a subsequent maternal collapse. A decision was made to return to theater for a re‐laparotomy and the on‐call surgeon was asked to re‐attend. Significant intra‐abdominal hemorrhage was noted. A maternal cardiac arrest occurred with cardiopulmonary resuscitation administered and massive transfusion protocol was commenced. Return of spontaneous circulation was established. Splenic vessel hemorrhage was suspected and an uncomplicated splenectomy and partial pancreatectomy were performed. Hemostasis was achieved. The abdomen was then packed with swabs. Total estimated blood loss was 8600 mL.

In total, 24 units of red cell concentrate, 16 g of fibrinogen, 10 Octaplas™ and 2 units of human albumin were given to the patient. She was transferred to a tertiary level 3 intensive care unit postoperatively. A re‐laparotomy was performed the next day where intra‐abdominal packs were removed. Her postoperative course was uneventful and she was discharged home on day 13.

Subsequent histological examination of the spleen and pancreas showed a “massively dilated vessel” at the splenic hilum, with immunohistochemical stains displayed lined endothelial cells and elastic stains with one or more layers of incomplete elastic lamina. The muscle fibers were incomplete and poorly orientated. This was interpreted as being in keeping with a SVA.

The patient was reviewed at 6 months postnatally and a full debrief was performed. Referral to the postnatal birth reflections clinic was organized for psychological support. No neurological impairment was noted from her cardiac arrest. From a neonatal perspective, her infant was meeting all major developmental milestones and there were no concerns regarding developmental delay.

## 3 RESULTS

Our systematic review details 10 reported cases of splenic vein rupture in pregnancy, which are summarized in Table 1.

**TABLE 1:** Summary of case reports found during the systematic review describing the rupture of the splenic vein in pregnancy.

Author (year)	Maternal age, gestational age	Comorbidities	Diagnosis	Presentation	Imaging performed with result, if reported	Definite management	Histological examination of splenic vein	Outcome: maternal and fetal
Kane et al.^a^, present study	30 years, G6, Para 4, 37 + 5 weeks	Recurrent Urinary tract infections	Splenic vein aneurysm rupture	Abdominal pain	Ultrasound (obstetric)	Laparotomy with splenectomy and partial pancreatectomy	Rupture of splenic vein aneurysm	Maternal survival, fetal survival
Marchi et al. (2020)^18^	32 years, G2, Para 1, 33 + 3 weeks	Nil noted	Spontaneous splenic vein rupture	Maternal collapse (hypotensive/tachycardic)	Ultrasound (obstetric)—“fetal bradycardia”	Laparotomy and splenectomy	No histopathologic evidence of aneurism was found	Maternal survival, fetal demise
Ajmal et al. (2012)^19^	Maternal age, gravidity or parity not recorded, 32 weeks	Left adnexal dermoid cyst	Spontaneous splenic vein rupture	Abdominal pain	Ultrasound (Obstetric)—“normal”	Laparotomy, cesarean section, sparing of the spleen	N/A	Maternal survival, fetal survival
Parpaglioni et al.^a^ (2009)^6^	28 years, G2, Para 1, 1 day postpartum after term delivery	Nil noted	Splenic vein aneurysm rupture	Maternal collapse (hypotensive/tachycardic), preceded by abdominal pain and distension	Trans‐abdominal ultrasound and CT‐abdomen/pelvis	Laparotomy with splenectomy and distal pancreatectomy	Splenic vein aneurysm	Maternal survival, fetal survival (as postnatal)
Turan et al. (2007)^21^	28 years, G2, Para1, 20 weeks (“5 months”)	Nil noted	Spontaneous splenic vein rupture	Maternal collapse	Not applicable	Not applicable	Rupture of splenic vein (0.5 cm in length) 3 cm from hilum of spleen	Maternal and fetal demise
Morelli et al.^a^ (2007)^24^	31 years, G3, Para 2, 36 weeks	Obesity	Splenic vein aneurysm rupture	Maternal collapse	Not applicable	Not applicable	Rupture of splenic vein saccular aneurysm	Maternal and fetal death
Madhaven et al. (1998)^7^	38 years, “multigravida”, 34 weeks	Nil noted	Spontaneous splenic vein rupture	Maternal collapse (hypotension/dyspnoeic)	Transabdominal‐ultrasound showing ‘free fluid’.	Laparotomy and splenectomy with oversewing of splenic vein tear	Not reported	Maternal survival, fetal demise
Wheelock et al. (1982)^13^	NA, NA, 34 weeks	NA	Spontaneous splenic vein rupture	NA	NA	NA	NA	Maternal survival, fetal demise
Newman et al. (1963)^20^	19 years, G1, Para 0, 34 weeks (“8 1/2 months”)	Nil noted	Spontaneous splenic vein rupture	Maternal collapse (hypotensive/tachycardic), preceded by abdominal pain	Nil	Laparotomy and splenectomy	Ruptured cavernous hemangioma of the splenic vein with marked peripancreatic hemorrhage and hematoma	Maternal demise (day 1 post‐op) and neonatal demise (1 h post‐delivery)
Eckerling (1962)^11^	27 years, G3, Para 2, 40 weeks (“9th month”)	Nil noted	Spontaneous splenic vein rupture, no discernible aneurysm	Maternal collapse (“no perceptible pulse rate, no blood pressure reading”). Severe abdominal pain preceding same	Nil	Laparotomy and splenectomy	Both macroscopic and microscopic examination of the splenic vein revealed no pathologic changes	Maternal survival, fetal demise
Shephard (1961)^12^	33 years, G5, Para 4, 28 weeks (“7 months”)	Nil noted	Spontaneous splenic vein rupture with inflammatory change, no discernible aneurysm	Abdominal pain which proceeded to maternal collapse	Nil	Laparotomy and splenectomy	Perforation of one of the branches of the splenic vein at hilum of spleen. Microscopic examination showed acute inflammatory reaction and fibrinoid alteration in some areas	Maternal survival, fetal demise

*Note:* We have included our case for comparison.

Abbreviations: G, gravidity; NA, not available/reported.

^a^ Cases of splenic vein aneurysm rupture.

Our review found spontaneous rupture of the splenic vein in eight cases with no reported evidence of aneurysm, with only two reporting rupture of a SVA. As regards the timing in relation to pregnancy of the splenic vein rupture, eight cases occurred in the third trimester, one in the second trimester, and one occurred post‐partum. Imaging preoperatively was performed in four cases, with only two noting a potential likelihood of hemoperitoneum.

Splenectomy was performed in five cases with a splenectomy and partial pancreatectomy in one case. Splenic conservation was performed in one case.

In relation to outcome, there were two cases of maternal and fetal survival, five cases where there was maternal survival with fetal demise, and three cases with both maternal and fetal demise.

## 4 DISCUSSION

We have described a case of a spontaneous rupture of a SVA in a women in her third trimester of pregnancy, causing hemodynamic compromise, necessitating emergent delivery of the infant by cesarean section followed by a splenectomy and distal pancreatectomy, resulting in a massive obstetric hemorrhage. There was both maternal and fetal survival. This is an exceedingly rare clinical entity, with only two other reports discussing SVA rupture. We have also conducted a systematic review of the literature in relation to splenic vein rupture and will compare and contrast this with our case.

The first case of a SVA was described in 1953^4^ with the first case in pregnancy described in 1959.^5^ Since this time, 10 cases in pregnancy have been described,^5^, ^6^, ^7^, ^8^, ^9^, ^10^, ^11^, ^12^, ^13^ with a significantly higher number of splenic artery aneurysms (SAAs) being reported.^14^ The incidence of SVA in pregnancy is unknown; however, the incidence of SAAs in pregnancy has been reported as 0.6 in 10 000 pregnancies.^14^ It is likely, given such few case reports, that the rupture of a SVA in pregnancy has a much lower incidence. Most SVAs are found incidentally, with patients not exhibiting any symptoms.^15^, ^16^ Surgical management is reserved for symptomatic SVA, with radiological surveillance being advocated for in those without symptoms.^17^

The origins of both SAAs and SVAs exhibit similarities despite their distinct incidence rates.^6^ These parallels encompass congenital factors such as local connective and elastic tissue insufficiency, as well as acquired causes like trauma, inflammation, and portal hypertension. In non‐pregnant females, SVA origins commonly involve portal hypertension, liver cirrhosis, chronic pancreatic inflammation, and vessel wall fragility.^15^ During pregnancy, SVA rupture can stem from acute pancreatic necrosis eroding the splenic vein,^5^ acute vessel wall inflammation,^12^ or splenic artery aneurysm rupture into the splenic vein.^10^ Some cases of splenic vein rupture defy clear explanation, leading to their categorization as “spontaneous” due to the absence of identifiable trauma or disease. In our case there were no risk factors for portal hypertension, with the patient having normal liver function testing during her in‐patient stay. From our review it is also evident that none of the cases had any reported significant comorbidities, with one case being subsequently followed up with no evidence of connective tissue disorder or hepatic dysfunction.^18^

Although SAAs and SVAs may arise from different etiologies, their clinical presentations, courses, complications, and management strategies likely overlap given that most have presented as acute hemodynamic compromise.,^3^, ^7^, ^11^, ^18^, ^19^, ^20^ which is demonstrated in our case. The differential diagnosis of SIAH in pregnancy includes visceral causes (hepatic, splenic, renal, and adrenal), gynecologic causes, coagulopathy‐related, and vascular causes (aneurysms). The definite management of suspected SIAH including SVA rupture with hemodynamic instability should be immediate resuscitative efforts following by emergent laparotomy. In cases where the patient is stable, emergent imaging such as computed tomography (CT) or magnetic resonance imaging (MRI) should be considered to ascertain the diagnosis with prompt treatment of the cause, once established. It should also be noted, however, that not all splenic vessel pathologies are aneurysmal^18^, ^21^ and therefore contrast imaging will not always give a diagnosis. Our unit is a stand‐alone obstetric hospital and, outside of regular working hours, only has access to point‐of‐care ultrasound. This case highlights the challenges faced by stand‐alone obstetric units, emphasizing the crucial need for coordinated care across hospital sites. Such coordination is especially important when emergent imaging, such as CT or MRI, is needed for an unwell patient. Of note, the 2019–2021 MBRRACE report has highlighted the fact that “women of reproductive age presenting to the emergency department collapse… should have a Focused Assessment with Sonography in Trauma (FAST) scan to exclude intra‐abdominal bleeding”.^22^ This, however, is dependent on the skill of the practitioner carrying out the ultrasound.^23^

Of note, in our case, the indication for emergent surgical management was due to a suspected obstetric cause of hemodynamic maternal and fetal compromise (i.e., an abruption) and an extrauterine cause was not suspected. Regardless of whether intra‐abdominal hemorrhage had been suspected, emergency delivery would still have been warranted given the finding of a fetal bradycardia. As acute abdominal pain in pregnancy has a wide differential, our case and subsequent systematic review demonstrate the difficulty that obstetricians face in ascertaining a diagnosis when both obstetric and non‐obstetric causes need to be considered. Our review demonstrated that, in some cases, extrauterine hemorrhage was suspected,^7^, ^11^ but in other cases it was not.^19^ In our case, intra‐abdominal hemorrhage was not initially suspected. This reiterates the need for maintaining a wide differential in an obstetric population for acute abdominal pain. In Table 2 we have listed the differential diagnosis that should be considered for acute non‐obstetric left‐sided abdominal pain in pregnancy.

**TABLE 2:** Differential diagnosis of non‐obstetric causes of left‐sided abdominal pain in pregnancy.

System	Diagnosis
Gastrointestinal	Constipation
	Gastritis
	Peptic ulcer disease
Biliary	Pancreatitis
Urinary	Urolithiasis
	Pyelonephritis
Splenic	Infarct
	Rupture
	Vessel rupture (artery/vein)
	Abscess
Other	Cardiac (myocardial infarction)
	Respiratory (pulmonary embolism, pneumothorax)

Our review has shown both significant maternal and fetal morbidity and mortality in relation to splenic vein rupture. There was only one other case reported where there was maternal and fetal survival where the fetus was still in utero during the event.^19^ Of note, this case did not have a preceding maternal collapse. Our case therefore highlights the potential positive outcome, with both maternal and fetal survival, when prompt surgical management is undertaken in the context of maternal collapse.

## 5 CONCLUSION

In conclusion, managing SIAH in pregnancy which includes splenic vein rupture presents unique challenges due to the need to balance the urgency of controlling hemorrhage, preventing maternal compromise, as well as preserving fetal viability. Prompt recognition, accurate diagnosis, and timely intervention are paramount to optimize maternal and fetal outcomes. However, the management approach must be individualized based on factors such as gestational age, hemodynamic stability, fetal status, and underlying etiology.

## AUTHOR CONTRIBUTIONS

DK, MG, CM, and CMM conceptualized the idea for the narrative review. DK, ET, RD, and JMK selected the articles and performed data extraction and cross‐checked the data. DK and CM wrote the first draft of manuscript with input from JD, CMM, ED, and JMC. JD, CMM, DK and JMC were all involved in the patient's care. All authors contributed to the writing of this article and approved the final version.

## FUNDING INFORMATION

No funding was received for this narrative review.

## CONFLICT OF INTEREST STATEMENT

The authors have no conflicts of interest.

## Data Availability

Data sharing is not applicable to this article as no new data were created or analyzed in this study.

## References

[1] Mazzocco MI , Donati S , Maraschini A , et al. Spontaneous hemoperitoneum in pregnancy: Italian prospective population‐based cohort study. Acta Obstet Gynecol Scand. 2022;101(11):1220‐1226. 36047477 10.1111/aogs.14431 PMC9812087

[2] Yang L , Liu N , Long Y . Intra‐abdominal hemorrhage during pregnancy: four case reports. World J Clin Cases. 2020;8(14):3074‐3081. 32775389 10.12998/wjcc.v8.i14.3074 PMC7385614

[3] Aung YY‐M , Berry C , Jayaram PR , Woon EV . Splenic artery aneurysm in pregnancy: a systematic review. Int J Gynecol Obstet. 2023;160(1):1‐11. 10.1002/ijgo.14278 35598155

[4] Loewenthal M , Jacob H . Aneurysm of the splenic vein; report of a case. Acta Med Orient. 1953;12(6):170‐173. 13091713

[5] Rahn J . Splenic vein rupture during pregnancy following acute pancreatic necrosis. Zentralbl Gynakol. 1959;81:1823‐1830. 14435869

[6] Parpaglioni R , Metta E , Zagari A , Celleno D . Spontaneous splenic vein aneurysm rupture in the puerperium. Int J Obstet Anesth. 2009;18(1):48‐51. 18684614 10.1016/j.ijoa.2008.01.019

[7] Madhavan P , Hegarty P , Akram M , Drumm J . Spontaneous rupture of the splenic vein in pregnancy. Ir Med J. 1998;91(2):64. 9617035

[8] Puder H . Thrombosis and rupture of the splenic vein in" acute obstetrical hypercoagulemic syndrome". Zentralblatt Fur Gynakologie. 1961;83:1370‐1375. 14489122

[9] Friebel HG . Rupture of the splenic vein and pancreatic necrosis in pregnancy. Zentralbl Gynakol. 1962;84:137‐145. 13959720

[10] Régent D , Hodez C , Bigard MA , Régent MC , Gaucher P , Roussel J . Splenic arterial aneurysm rupturing into the splenic vein. A rare cause of acute fortal hypertension in the post‐partum period (author's transl). J Radiol Electrol Med Nucl. 1977;58(2):151‐154. 557548

[11] Eckerling B , Goldman JA , Yado S . Spontaneous rupture of the splenic vein in pregnancy with massive retro‐ and intraperitoneal hemorrhage. Am J Surg. 1962;103:836‐839. 13889097 10.1016/0002-9610(62)90536-6

[12] Shepard D , Foster CE . Spontaneous rupture of the splenic vein during pregnancy. Ann Surg. 1961;153(6):1029‐1032. 17859665 10.1097/00000658-196106000-00022 PMC1614010

[13] Wheelock JB . Spontaneous splenic vein rupture in pregnancy: case report. Mil Med. 1982;147(1):64. 6806687

[14] Ornaghi S , Crippa I , di Nicola S , et al. Splenic artery aneurysm in obstetrical patients: a series of four cases with different clinical presentation and outcome. Int J Gynecol Obstet. 2022;159(2):474‐479. 10.1002/ijgo.14133 35122689

[15] Torres G , Hines GL , Monteleone F , Hon M , Diel J . Splenic vein aneurysm: is it a surgical indication? J Vasc Surg. 1999;29(4):719‐721. 10194502 10.1016/s0741-5214(99)70320-4

[16] Gharabaghi MA , Yazdi NA , Abrishami Z , Amini S . Splenic vein aneurysm. BMJ Case Rep. 2012;2012:bcr1020115007. 10.1136/bcr.10.2011.5007 PMC3291015 22665464

[17] Shimoda M , Kubota K , Sakuma A , Hogami T , Yamaguchi H , Tagaya N . Intra‐abdominal hemorrhage due to rupture of a splenic vein aneurysm: a case report. J Gastrointest Surg. 2003;7(5):683‐686. 12850682 10.1016/s1091-255x(03)00028-3

[18] Marchi L et al. Spontaneous splenic vein rupture with massive hemoperitoneum during the third trimester of pregnancy. Asploro J Biomed Clin Case Rep. 2020;4(1):1‐5.

[19] Ajmal M . Spontaneous splenic vein bleeding during pregnancy: consequences of a missed diagnosis. J Anesth. 2012;26(6):959‐960. 22832838 10.1007/s00540-012-1450-2

[20] Newman B , Gaal GZ , Jackson E . Spontaneous rupture of the splenic vein during pregnancy. South Med J. 1963;56:11‐13. 13938257 10.1097/00007611-196301000-00002

[21] Turan N , Oghan F , Boran T . Spontaneous rupture of splenic vein in a pregnant woman during a religious ritual. J Forensic Leg Med. 2007;14(7):440‐443. 17720597 10.1016/j.jflm.2006.12.005

[22] Knight M , Bunch K , Felker A . Saving Lives, Improving Mothers' Care Core Report—Lessons Learned to Inform Maternity Care from the UK and Ireland Confidential Enquiries into Maternal Deaths and Morbidity 2019–21. National Perinatal Epidemiology Unit, University of Oxford; 2023.

[23] Wiles R , Hankinson B , Benbow E , Sharp A . Making decisions about radiological imaging in pregnancy. BMJ. 2022;377:e070486. 35470230 10.1136/bmj-2022-070486 PMC9036096

[24] Morelli L , Pusiol T , Parolari AM , Piscioli I . First report of an unusual, rare complication of pregnancy: rupture of multiple lienal vein aneurysms. Arch Gynecol Obstet. 2007;275(4):275‐277. 16924509 10.1007/s00404-006-0231-2

